# Gender differences in association of urbanization with psychological stress in Chinese adults: A population-based study

**DOI:** 10.3389/fpubh.2022.1022689

**Published:** 2022-11-14

**Authors:** Dianjiang Li, Yuhui Ruan, Qi Kang, Chao Rong

**Affiliations:** ^1^Department of Social Medicine and Health Education, School of Public Health, Nanjing Medical University, Nanjing, China; ^2^Research Center for Social Risk Governance for Major Public Health Events, Nanjing Medical University, Nanjing, China; ^3^School of Politics and Public Administration, Soochow University, Suzhou, China; ^4^Institute of Public Health, Soochow University, Suzhou, China; ^5^Department of Health Policy Research, Shanghai Health Development Research Center (Shanghai Medical Information Center), Shanghai, China; ^6^Department of Health Service and Management, School of Humanities and Management, Zhejiang Chinese Medical University, Hangzhou, China

**Keywords:** China, urbanization, community, psychological stress, multilevel analysis

## Abstract

**Objective:**

To investigate the gender-specific associations between exposure to urbanization and psychological stress in China experiencing rapid urbanization.

**Methods:**

Data were obtained from the 2015 China Health and Nutrition Survey. A total of 4,388 men and 5,098 women aged at least 18 years were obtained from 288 communities across 12 provinces and municipalities. Tertiles of the urbanization index, summarizing 12 urbanization dimensions at the community level, were used to define low, medium, and high levels of urbanization. The psychological stress was measured based on the 10-item Perceived Stress Scale. The gender-stratified multilevel analysis (Level-1: Individuals, Level-2: Communities, and Level-3: provinces/municipalities) was used to estimate the association between exposure to urbanization and psychological stress.

**Results:**

After controlling for age, education status, marital status, work status, household income per capita, current smoking, alcohol drinking, sleep duration, BMI, and chronic conditions, the urbanization index was negatively associated with psychological stress in women (*P*_trend_ = 0.017) but not men (*P*_trend_ = 0.476). More specifically, a one-standard deviation increase in the score of community population density (β = −0.329, *P* = 0.329), modern markets (β = −0.247, *P* = 0.044), education (β = −0.448, *P* = 0.002), and housing (β = −0.380, *P* = 0.005) was negatively associated with psychological stress only in women, separately.

**Conclusion:**

Our data revealed that living in the most urbanized communities is associated with lower levels of psychological stress for women but not men. Thus, this study can help empower decision-makers to accurately target vulnerable communities and plan effective strategies to address psychological outcomes.

## Introduction

Urbanization involves the change in size, density, and heterogeneity in places (1), and is widely recognized as a driver of the main health-relevant changes to humanity (2). Currently, more than 55% of the global population is living in urban areas. This proportion will increase to nearly 70% by 2050, with at least 50% of the population living in cities of more than 500,000 inhabitants (3). Rapid urbanization generated opportunities for improved sanitation, infrastructure, education, communication networks, and better health care services. Nevertheless, it resulted in traffic congestion, sedentary lifestyles, and environmental pollution (4–6). These factors can independently and synergistically influence individuals' health (7–9). A better understanding of their relationships may help us identify interventions that effectively promote health status in countries undergoing rapid urbanization.

China has witnessed unprecedented urbanization and associated rural depopulation over the past 40 years, along with a huge increase in the total population (10, 11). The rapid migration and urbanization process in China has led to pronounced changes in community composition and its characteristics (12), evoking great scholarly interest in the effects of urbanization on mental health for Chinese people (13–18). Most studies suggested an inverse relationship between urbanization and mental health (14, 16, 19), with urban living related to a lower prevalence of developing mental health problems (such as schizophrenia, dementia, cognitive impairment, and mood disorders) (14, 16, 17). Especially residence in highly urbanized areas was associated with lower depressive symptoms (20). However, few studies examined the association between exposure to urbanization and psychological stress in China. Psychological stress refers to the real or perceived environmental demands that exceed an individual's adaptive capacity in daily life (21, 22) and is considered the most well-established risk factor for mental disorders (23–25). It is, therefore, vital to obtain a comprehensive view of the complex and evolving relationship between urbanization and psychological stress for sustainable urbanization that can be deployed to protect mental health.

Moreover, rapid urbanization affects women and men in fundamentally different patterns (8, 26). Women and men may perceive, be exposed to, and respond differently to a growing urbanized environment (27). Meanwhile, gender affects each element in the stress process as much as the input by deciding whether a situation will be stressful at the output (28). Previous epidemiological studies have revealed that women are more likely to suffer from higher psychological stress than men (29–31) and develop greater mental health problems (14, 16, 17). Therefore, these observations suggest the possibility of gender-specific differences in the effects of urbanization on psychological stress. Thus, this study aimed to use data from the China Health and Nutrition Survey (CHNS) to explore the gender-specific association between urbanization and psychological stress, with a multidimensional index assessment at the community level.

## Materials and methods

### Study population and design

This study used data from CHNS, a longitudinal study that began in 1989 and has been repeated every 2–4 years through 2015 in 10 waves. The CHNS used a multistage, random cluster process to draw the sample surveyed in nine provinces (Liaoning, Heilongjiang, Jiangsu, Shandong, Henan, Hubei, Hunan, Guangxi, and Guizhou) (32). The sample was designed to represent rural and urban areas of varying geography, economy, and public resources, and to focus on the overall health during urbanization and economic transitions (32). In 2011, three municipalities (Beijing, Chongqing, and Shanghai) directly under the Central Government were added to the study. The scientific rationale and design of the CHNS have been reported in detail elsewhere (33).

For the analysis, we included 20,899 participants over 18 years old from the 10th survey of the CHNS carried out in 2015, which measured psychological stress for the first time. Participants who had missing information on urbanization (*n* = 36), psychological stress (*n* = 1,413), or control variables including household income (*n* = 7,345), current smoking (*n* = 969), alcohol drinking (*n* = 4), sleep duration (*n* = 125), height (*n* = 1,512), or chronic conditions (*n* = 9) were excluded. Thus, 9,486 subjects (4,388 men and 5,098 women) from 288 communities across 12 provinces and municipalities were included in the final analysis.

### Assessment of urbanization

We evaluated the urbanization level of the 288 sampled communities using an urbanization index developed by Jones-Smith and Popkin (34). Specifically, the urbanization index was applied to 12 multidimensional components at a community level: communications (e.g., television, mobile, post, and cinema), population density, economic activity, housing (i.e., availability of electricity, indoor tap water, and flushing toilets), traditional markets (i.e., types, distances, and business hours of food and fuel markets), modern markets (i.e., the quantity of supermarkets and modern eating establishments), social services, transportation, education (i.e., average educational level among adults above 21 years old), diversity (i.e., community variance in education and income levels), health infrastructure, and sanitation. Each component was scaled from 0 to 10, weighted equally in the overall index, and added together for an overall maximum possible score of 120. Higher scores reflected more urban characteristics across multiple domains. The urbanization index was explicitly developed for the CHNS and had adequate reliability and validity in previous studies (32, 35). The indicators measuring the proportion of households were derived from the household responses and the remaining indicators were collected from the community-level survey offered to community officials. The detailed construction procedure has been described elsewhere (34, 36, 37). Similar to prior studies, the urbanization index is categorized into tertiles representing low (<63.52), medium (63.52–84.07), and high (>84.07) urbanization levels.

### Assessment of psychological stress

The Perceived Stress Scale-10 (PSS-10) was designed to measure the degree to which situations in one's life are appraised as stressful (38) and has been verified to assess psychological stress in a large community-based general population in China (24, 39). The PSS-10 is divided into two subscales: negative and positive. The negative subscale assesses the lack of control in life and negative affective reactions (perceived distress), while the positive subscale measures the ability to cope with current stressors (coping capacity) (38, 40). The participants rated each item on a 5-point Likert scale ranging from “0 = never” to “4 = very often. Positively framed questions (items 6, 7, 9, and 10) were reverse scored (“4 = never” to “0 = very often”), and all 10-item scores were then summed to create a total score. The total score ranged from 0 to 40 (Cronbach's α = 0.744 in the present study), with higher scores indicating higher levels of psychological stress.

### Assessment of control covariates

Trained interviewers collected individual information on sociodemographics, lifestyle parameters, and medical history with a structured questionnaire. The education status was divided into three categories (primary school and below, junior school, or senior school and above), and the marital status was categorized as married or others (including divorced, widowed, separated, or never married). The work status was divided into two categories (yes or no). The annual household income per capita (yuan) was calculated as the sum of the self-reported annual income of all adult family members divided by household size. Lifestyle factors included in the analysis were current smoking (yes or no), alcohol drinking (yes or no), and sleeping. Sleep duration was obtained by asking how many hours a person sleeps every day, including daytime and night time, and was categorized as ≤6 h, 7–8 h, or ≥9 h. The body mass index (BMI) was calculated as the weight (kg) divided by height (m) squared and was divided into four categorical groups based on the criteria recommended by the Working Group on Obesity in China (41): underweight (< 18.5 kg/m^2^), normal (18.5–23.9 kg/m^2^), overweight (24.0–27.9 kg/m^2^), or obesity (≥28.0 kg/m^2^). Chronic conditions were measured by asking the respondents whether they had been diagnosed with chronic diseases (including hypertension, diabetes mellitus, stroke, myocardial infarction, or cancer) by professional doctors (yes or no).

### Statistical analysis

Given that the continuous variables were of non-normal distribution, the results were presented as medians (P_25_, P_75_) for continuous variables and numbers (percentages) for categorized variables. The characteristics between men and women were compared using the Wilcoxon rank-sum test or Chi-squared test, as appropriate. Due to the prominent hierarchical characteristics of the data, individuals (level 1) nested within the community (level 2), and province/municipality (level 3) (42, 43), we used a gender-stratified multilevel (three-level) linear regression model to investigate the association between the community-level urbanization index and psychological stress. We developed two models, model 1 was unadjusted, and model 2 was adjusted for individual-level variables including age (continuous), education status, marital status, work status, household income per capita (continuous), current smoking, alcohol drinking, sleep duration, BMI, and chronic conditions. In addition, we examined whether each of the 12 urbanization components was separately associated with psychological stress. Given collinearity between the 12 urbanization components and to better understand the pathways linking exposure to urbanization and psychological stress, we conducted a separate analysis. We incorporated each of the 12 urbanization components [in one-standard deviation (SD) increase increments] in the model while adjusting for the covariates included in model 2 and stratifying by gender.

The statistical significance was determined at a two-sided *P*-value < 0.05 level, and all statistical analyses were conducted using SAS 9.4 (SAS Institute Inc., Cary, NC, USA) for Windows.

## Results

### Characteristics of the participants

Table 1 reports the characteristics of the 9,486 participants aged 18–99. The median (P_25_, P_75_) age of the total population was 53 (43, 63) years, with men having a higher median (P_25_, P_75_) age than women [54 (44, 64) vs. 53 (42, 63)]. Men were more likely to be educated, married, employed, and have higher median household income per capita, whereas women smoked and drank less and had higher levels of obesity. Men more frequently reported a history of chronic conditions than women. The median (P_25_, P_75_) score of PSS-10 was 16 (12, 19) for men and 17 (13, 19) for women.

**Table 1:** Characteristics of the study population.

**Characteristics**	**Overall**	**Men**	**Women**	***P*-value**
No. of participants	9,486	4,388	5,098	
Age (years)*	53 (43, 63)	54 (44, 64)	53 (42, 63)	0.003
**Education status**, ***n*** **(%)**
Primary school and below	3,064 (32.3)	1,136 (25.89)	1,928 (37.82)	< 0.001
Junior school	5,150 (54.29)	2,601 (59.28)	2,549 (50.00)	
Senior school and above	1,272 (13.41)	651 (14.84)	621 (12.18)	
**Marital status**, ***n*** **(%)**
Married	8,328 (87.79)	3,935 (89.68)	4,393 (86.17)	< 0.001
Others	1,158 (12.21)	453 (10.32)	705 (13.83)	
**Work status**, ***n*** **(%)**
No	4,913 (51.79)	1,867 (42.55)	3,046 (59.75)	< 0.001
Yes	4,573 (48.21)	2,521 (57.45)	2,052 (40.25)	
Household income per capita (10,000 yuan)*	1.77 (0.83, 3.20)	1.81 (0.87, 3.23)	1.73 (0.80,3.16)	0.012
**Current smoking**, ***n*** **(%)**
No	7,302 (76.98)	2,315 (52.76)	4,987 (97.82)	< 0.001
Yes	2,184 (23.02)	2,073 (47.24)	111 (2.18)	
**Alcohol drinking**, ***n*** **(%)**
No	6,892 (72.65)	2,066 (47.08)	4,826 (94.66)	< 0.001
Yes	2,594 (27.35)	2,322 (52.92)	272 (5.34)	
**Sleep duration (hours)**, ***n*** **(%)**
≤ 6	1,161 (12.24)	533 (12.15)	628 (12.32)	0.967
7–8	1,646 (17.35)	763 (17.39)	883 (17.32)	
≥9	6,679 (70.41)	3,092 (70.46)	3,587 (70.36)	
**BMI**, ***n*** **(%)**
Underweight	1,363 (14.37)	621 (14.15)	742 (14.55)	< 0.001
Normal	3,422 (36.07)	1,670 (38.06)	1,752 (34.37)	
Overweight	432 (4.55)	178 (4.06)	254 (4.98)	
Obesity	4,269 (45.00)	1,919 (43.73)	2,350 (46.10)	
**Chronic conditions**, ***n*** **(%)**
No	7,540 (79.49)	3,439 (78.37)	4,101 (80.44)	0.013
Yes	1,946 (20.51)	949 (21.63)	997 (19.56)	
PSS-10*	16 (12,19)	16 (12,19)	17 (13,19)	< 0.001

^*^Data were presented as median (P_25_, P_75_).

BMI, body mass index; PSS-10, Perceived Stress Scale-10.

### Urbanization index and psychological stress

Table 2 reports the multilevel-adjusted associations between the urbanization index and psychological stress. In men, no significant association between urbanization index and psychological stress was observed in model 1 (*P*_trend_ = 0.209) and model 2 (*P*_trend_ = 0.476). In women, model 1 presented a higher urbanization index associated with lower psychological stress (*P*_trend_ = 0.001). Regarding model 2, the association reduced but remained statistically significant when adjusting for age, education status, marital status, work status, household income per capita, current smoking, alcohol drinking, sleep duration, BMI, and chronic conditions (*P*_trend_ = 0.017). Specifically, women were significantly more likely to report a lower psychological stress medium (β = −0.661, *P* = 0.034) and high (β = −0.775, *P* = 0.014) levels of urbanization than low urbanization in model 2.

**Table 2:** Multilevel linear regression for the association between urbanization index and psychological stress, stratified by gender.

**Urbanization**	Model 1*				**Model 2** ^**†**^			
**index**	**β**	**SE**	***P*-value**	***P*_trend_**	**β**	**SE**	***P*-value**	***P*_trend_**
**Men**
Low	1.000 (ref.)	0.9	1.000 (ref.)	0.6
Medium	−0.005	0.316	0.987		0.044	0.316	0.890	
High	−0.377	0.314	0.230		−0.219	0.321	0.496	
**Women**
Low	1.000 (ref.)	0.1	1.000 (ref.)	0.7
Medium	−0.777	0.309	0.012		−0.661	0.311	0.034	
High	−0.996	0.306	0.001		−0.775	0.316	0.014	

^*^Model 1: unadjusted.

^†^Model 2: adjusted for age (continuous), education status (primary school and below, junior school, or senior school and above), marital status (married or others), work status (yes or no), household income per capita (continuous), current smoking (yes or no), alcohol drinking (yes or no), sleep duration ( ≤6, 7–8, or ≥9 h), BMI (underweight, normal, overweight, or obesity), and chronic conditions (yes or no).

SE, standard error.

### Urbanization components and psychological stress

The multilevel-adjusted associations of urbanization components (per one-SD increase) and psychological stress are presented in Table 3. In men, a one- SD increase in the score of education and housing at the community level was separately associated with lower psychological stress in model 1, but the associations were not statistically significant when controlling for the variables in Model 2. In women, model 1 showed that a one-SD increase in the score of community population density, social services, modern markets, transportation, economic activity, education, housing, and sanitation was separately associated with lower psychological stress. Further adjustment for age, education status, marital status, work status, household income per capita, current smoking, alcohol drinking, sleep duration, BMI, and chronic conditions in Model 2, the increase in the score of community population density (β = −0.329, *P* = 0.329), modern markets (β = −0.247, *P* = 0.044), education (β = −0.448, *P* = 0.002), and housing (β = −0.380, *P* = 0.005) were still inversely associated with psychological stress, separately.

**Table 3:** Multilevel linear regression for the association between urbanization components and psychological stress, stratified by gender.

**Urbanization components**	Model 1*			**Model 2** ^**†**^		
	**β^‡^**	**SE**	***P*-value**	**β^‡^**	**SE**	***P*-value**
**Men**
Communications	−0.247	0.129	0.056	−0.206	0.130	0.114
Population density	−0.115	0.140	0.413	−0.075	0.142	0.597
Social services	−0.242	0.125	0.052	−0.198	0.125	0.114
Traditional markets	−0.168	0.125	0.179	−0.123	0.125	0.323
Modern markets	−0.120	0.123	0.331	−0.074	0.124	0.551
Transportation	−0.064	0.122	0.599	−0.036	0.122	0.771
Economic activity	−0.140	0.131	0.287	−0.096	0.132	0.470
Education	−0.334	0.139	0.016	−0.239	0.146	0.101
Diversity	−0.243	0.128	0.058	−0.204	0.128	0.112
Health infrastructure	0.001	0.125	0.992	0.021	0.124	0.864
Housing	−0.313	0.133	0.019	−0.261	0.135	0.053
Sanitation	−0.152	0.126	0.228	−0.126	0.127	0.321
**Women**
Communications	−0.215	0.128	0.093	−0.137	0.128	0.285
Population density	−0.387	0.137	0.005	−0.329	0.140	0.019
Social services	−0.284	0.123	0.021	−0.235	0.124	0.058
Traditional markets	−0.223	0.124	0.071	−0.154	0.124	0.213
Modern markets	−0.327	0.120	0.007	−0.247	0.123	0.044
Transportation	−0.250	0.120	0.037	−0.206	0.120	0.086
Economic activity	−0.290	0.129	0.024	−0.223	0.130	0.087
Education	−0.562	0.134	< 0.001	−0.448	0.142	0.002
Diversity	−0.170	0.126	0.178	−0.121	0.126	0.339
Health infrastructure	0.010	0.123	0.935	0.044	0.122	0.719
Housing	−0.460	0.130	< 0.001	−0.380	0.134	0.005
Sanitation	−0.308	0.125	0.014	−0.237	0.127	0.061

^*^Model 1: unadjusted.

^†^Model 2: adjusted for age (continuous), education status (primary school and below, junior school, or senior school and above), marital status (married or others), work status (yes or no), household income per capita (continuous), current smoking (yes or no), alcohol drinking (yes or no), sleep duration (≤6, 7–8, or ≥9 h), BMI (underweight, normal, overweight, or obesity), and chronic conditions (yes or no).

^‡^β co-efficient representing the effect of a one-standard deviation increase in the component score.

SE, standard error.

## Discussion

To our knowledge, this study is the first to test potential gender differences in the association of urbanization with psychological stress in the CHNS data using multilevel analyses. Our gender-specific analysis showed that men and women had different patterns in the association between the urbanization index and psychological stress. By further analyzing the results, women appeared to be more sensitive to the urbanization components than men. Moreover, four urbanization components were separately and negatively associated with the levels of psychological stress in women levels after controlling for potential confounders.

This study found that the urbanization index was negatively associated with psychological stress in women but not men. Women in the most urbanized communities were likely to have lower levels of psychological stress than those in the low urbanized communities. No association was found in men, and the results for both genders remained consistent after adjusting for age, education status, marital status, work status, household income per capita, current smoking, alcohol drinking, sleep duration, BMI, and chronic conditions. Similar to this work, prior studies have reported that living in urban areas was associated with a lower suicide rate, with a consistently lower rate in women than in men (44, 45). One potential explanation for the observed gender difference may be partly that social roles determine the range of potentially stressful experiences of women and men (28). In China, women are equally stressed by work and family, whereas men are more vulnerable to the psychological impact of work roles than family roles (46). During China's urbanization, urban women, in contrast to rural women, are more likely to strive to become financially independent by doing more market work and less housework (47, 48) and eventually win better happiness (48). In addition, the mechanisms for the observed gender differences may also be related to the different coping strategies of men and women. This is explained by studies showing that women tend to use adaptive coping skills more and are more likely to seek social support than men (49–53). For example, women tend to be relatively more willing and able to access neighborhood social resources when faced with stressful life experiences (20). In contrast, men resist receiving or seeking formal support but are more willing to accept social support from families (54). Distinct from western countries, China's urbanization was greatly intervened by government policies (55, 56) and might promote neighborhood social capital (20, 56), increasing access to social networks and supports, regulating unhealthy behaviors, and promoting adaptive coping mechanisms associated with better psychological outcomes (57).

This study first examined the pathways linking 12 components of urbanization and psychological stress. It demonstrated that one SD increase in the score of community population density, modern markets, education, and housing was negatively associated with psychological stress only in women after controlling for age, education status, marital status, work status, household income per capita, current smoking, alcohol drinking, sleep duration, BMI, and chronic conditions. Living in crowded communities is associated with decreased psychological stress since population density is one of the most widely used proxies for access to medical and mental health professionals (58, 59). Therefore, mental health service providers tend to be heavily concentrated in densely populated urban areas (60). Melis et al. found that high population density contributed to a reduced risk of depression, especially for women (61). Additionally, greater access to modern markets was associated with lower levels of psychological stress. Living in a community with easier access to modern markets promotes access to healthy foods, resulting in lower life stress (62) and better happiness (63, 64). Brown et al. reported that greater accessibility and shopping in modern supermarkets were associated with better self-rated health (64, 65). Education at a community level was inversely associated with psychological stress because the neighborhood educational attainment was considered “collective human capital” (66) and “collective efficiency” (67), which may benefit the residents' health more than the sum of their efforts (68). Wight et al. indicated that living in a low-education area was associated with low cognitive function, net of individual characteristics, including individual-level education (69). Noteworthy, a house is a crucial family asset and is regarded as a potent status symbol in China (70). This study found that better housing conditions were inversely associated with psychological stress, consistent with previous studies, showing that inadequate housing conditions are associated with worsening mental health (i.e., stress, anxiety, and depression) (71–73). Although pathways have been proposed to elucidate this association, future studies are required to validate the role of the other specific urbanization elements in improving psychological outcomes.

The strengths of this study were that the multicomponent urbanization index could capture heterogeneity in various services and infrastructures across different urbanized communities. We also evaluated how the pathways linking urbanization and psychological stress vary by gender, demonstrating a statistically significant association for women. The findings can help guide decision-makers to accurately target vulnerable communities and formulate effective policies to address psychological outcomes in China and other recently urbanized countries. However, there are several limitations to this study. First, the CHNS was not nationally representative, but encompassed communities from 12 different provincial regions in the northeast, central, and south China (27). This diversity makes this analysis possible (74). Second, the study was a cross-sectional design, limiting causality interpretations. Healthier people were more likely to migrate to more urbanized communities in search of better living conditions (20, 27, 75) because of the so-called healthy migrant effect (76). Third, this study decomposed urbanization and revealed that specific elements substantially affect psychological stress. Nevertheless, these single associations might be due to other factors associated with urbanization (77). Last, although measuring psychological stress in the CHNS has been validated (24, 39), it represents a self-reported perception of stress and is not an objective measurement. Future studies should attempt to replicate our analyses by measuring biological stress markers, such as cortisol levels (78–80).

## Conclusion

The current study in a large sample of Chinese adults showed that a higher urbanization index was negatively associated with psychological stress in women but not men. More specifically, one SD increase in the score of community population density, modern markets, education and housing was separately and negatively associated with psychological stress only in women after controlling for potential confounders. These findings provide valuable insights that help guide decision-makers to accurately target vulnerable communities and allocate public resources to address psychological outcomes in the case of rapid urbanization.

## Data availability statement

The datasets presented in this study can be found in online repositories. The names of the repository/repositories and accession number(s) can be found at: https://www.cpc.unc.edu/projects/china.

## Ethics statement

The studies involving human participants were reviewed and approved by the Institutional Review Committees of the Institute for Nutrition and Health of Chinese Center for Disease Control and Prevention and the University of North Carolina at Chapel Hill, USA. The patients/participants provided their written informed consent to participate in this study.

## Author contributions

DL conceived and designed the study, drafted the report, and received the final version for publication. YR conducted the statistical analysis. QK and CR revised the manuscript. All authors checked the article and approved it for publication.

## Funding

This work was supported by the National Natural Science Foundation of China (Project No. 71974101), the Humanities and Social Science Research Project of Soochow University (Project No. NH33716122), and the Youth Interdisciplinary Research Project in Humanities and Social Sciences of Soochow University (Project No. NH33714622).

## Conflict of interest

The authors declare that the research was conducted in the absence of any commercial or financial relationships that could be construed as a potential conflict of interest.

## Publisher's note

All claims expressed in this article are solely those of the authors and do not necessarily represent those of their affiliated organizations, or those of the publisher, the editors and the reviewers. Any product that may be evaluated in this article, or claim that may be made by its manufacturer, is not guaranteed or endorsed by the publisher.
